# Pathophysiology of intracranial hypertension in cryptococcal meningoencephalitis

**DOI:** 10.1128/mbio.00312-26

**Published:** 2026-04-13

**Authors:** Arie Van Wieren, Arturo Casadevall

**Affiliations:** 1 Department of Molecular Microbiology and Immunology, Johns Hopkins Bloomberg School of Public Health, Johns Hopkins University 1466, Baltimore, Maryland, USA; Vallabhbhai Patel Chest Institute, Delhi, India

**Keywords:** *Cryptococcus neoformans*, cryptococcal meningitis, mycology, intracranial hypertension, intracranial pressure, pathogenesis, host-pathogen interactions

## Abstract

Cryptococcal meningoencephalitis (CME) is a major cause of death and disability, and intracranial hypertension is a leading, treatable contributor to mortality and neurologic sequelae. Across CME cohorts, markedly elevated cerebrospinal fluid (CSF) opening pressure is common and often occurs despite minimal ventriculomegaly or diffuse edema on neuroimaging. This review synthesizes clinical, microbiological, imaging, pathological, and experimental evidence to define priorities for mechanistic research. Intracranial pressure (ICP) physiology predicts that once intracranial compliance is exhausted, small volume changes can produce rapid pressure increases, making CSF dynamics central to many intracranial hypertension syndromes. In CME, the frequent, rapid improvement after therapeutic CSF drainage, followed by pressure re-accumulation, supports a CSF outflow-limited mechanism for ICP. Convergent observations, including correlations between opening pressure and fungal/capsular polysaccharide burden and postmortem localization of organisms and polysaccharide at candidate CSF efflux sites, support a model of increased CSF outflow resistance. Potential modifiers include cryptococcal phenotypes (e.g., capsule size/architecture, aggregation), host immune and osmotic states, and disruption of perivascular (“glymphatic”) transport that may alter clearance and compliance. Alternative dominant mechanisms (e.g., mass effect, obstructive hydrocephalus, venous sinus thrombosis, or inflammatory edema in immune reconstitution inflammatory syndrome/post-infectious inflammatory response syndrome) likely account for a minority of cases but remain clinically important. Current ICP control relies on invasive CSF drainage, and empiric pharmacologic approaches have not translated well, meaning progress will depend on both clinical and basic science research that link fungal and host factors to ICP trajectories, quantify efflux-site burden, directly measure outflow resistance, and explore adjunctive therapeutics that address CSF efflux and fungal clearance.

## INTRODUCTION

Elevated intracranial pressure (ICP) is a leading cause of mortality and long-term neurologic morbidity in cryptococcal meningoencephalitis (CME) (1, 2). This phenomenon sets cryptococcal meningoencephalitis apart from other fungal meningitides and thus reflects the unique properties of *Cryptococcus neoformans* and the host response to infection. Among pathogenic eukaryotic microbes, *C. neoformans* is unique in having a large polysaccharide capsule that surrounds a yeast-sized cell and in copiously shedding capsular polysaccharide into tissues, including the cerebrospinal fluid (CSF), where it is measured diagnostically as cryptococcal antigen (3, 4).

CME is a major cause of death and disability worldwide, particularly among immunocompromised populations (5, 6). Despite optimization of antifungal drug combinations, mortality remains high in part because intracranial hypertension remains difficult to manage (7). Across cohorts, elevated CSF opening pressure (often defined as >25 cmH_2_O) is common (50%–70% of patients) and is associated with serious complications, including early death, vision loss, seizures, and other neurologic sequelae (8–13). A defining feature of intracranial hypertension in CME is its transient responsiveness to CSF removal: therapeutic lumbar punctures (LPs) can improve symptoms and are associated with improved survival, but ICP elevations frequently reoccur, necessitating repeated drainage (2, 10, 14). CSF removal via serial LPs remains an invasive procedure that is limited in low-resource settings, while trials of adjunctive therapeutics to lower ICP have produced disappointing results, likely because the underlying biology of this microbe-host interaction has yet to be thoroughly explored (15).

In this review, we update prior frameworks by synthesizing clinical, microbiological, imaging, pathological, and experimental evidence to evaluate mechanisms underlying intracranial hypertension in CME and to identify priorities for future research (15, 16). We first outline fundamental ICP pathophysiology and temporal patterns of ICP elevation in CME. Next, we review CSF pathway anatomy and how CME pathology overlaps with these sites. We then evaluate the convergent evidence that supports an increased CSF outflow-resistance model as the dominant mechanism while considering modifying microbial and host characteristics as well as alternative processes that are likely to account for a minority of cases. Finally, we propose testable directions for future study.

## FRAMING TERMS AND PHYSIOLOGY

In approaching the topic of intracranial hypertension in CME, it is important to define four related terms. (i) ICP is the pressure within the cranial vault. (ii) CSF opening pressure is the manometer-measured pressure obtained during LP; it is a commonly used clinical surrogate for ICP but can diverge in uncommon situations (e.g., large focal mass effect). (iii) Intracranial hypertension is a clinical syndrome of sustained/pathologic ICP elevation. (iv) Hydrocephalus is altered CSF dynamics accompanied by ventricular enlargement (ventriculomegaly) on neuroimaging. These distinctions are particularly important in cryptococcal meningoencephalitis, where markedly elevated CSF opening pressure commonly occurs without an increase in ventricle size or diffuse cerebral edema (17).

Brain tissue, CSF, and blood volume influence pressure within the rigid skull, with current models recognizing that ICP arises from a system of intracranial dynamics rather than a purely static volume-pressure relationship (18, 19). The classic Monro–Kellie doctrine posits that because total intracranial volume is effectively fixed within the cranium, an increase in one component must be offset by a decrease in another, or ICP will rise (18). An updated framework, Monro–Kellie 4.0, emphasizes that ICP elevation is determined by the compensatory response of the system, which depends on dynamic processes such as intracranial compliance, venous outflow, cerebrovascular autoregulation, and glymphatic function (19). Intracranial blood volume (primarily the venous compartment) and CSF are the components most able to be shunted out to accommodate an increase in intracranial content (20). Once this compensatory reserve is exhausted, small volume increases can cause steep rises in ICP (18).

Sustained ICP elevation can lead to brain herniation and death and/or reduced cerebral blood flow with ischemia and infarction (21). Zhu et al. reported a brain herniation incidence of 19.5% in HIV-negative CME patients, and time-varying cerebral perfusion pressure outside the normal range has been associated with higher 2-week mortality for patients with CME, emphasizing the clinical consequences of ICP elevation (12, 22).

## MECHANISTIC OBSERVATIONS FROM THE BEDSIDE

Neuroimaging manifestations of CME are heterogeneous and often subtle, limiting simple one-to-one mapping between a radiographic abnormality and the mechanism of intracranial hypertension. On CT imaging, findings are frequently non-specific, and normal scans are seen in a large proportion of patients (reported up to 47%), while MRI provides improved detection of abnormalities (17, 23, 24). Hydrocephalus/ventriculomegaly is relatively uncommon (2%–18%) in CME imaging cohorts despite consisting of a population where high opening pressures are common (8, 17, 25–27). Similarly, across published imaging cohorts and on postmortem analysis, diffuse/global edema is rare or not seen, suggesting generalized swelling is uncommon and not a primary mechanism of ICP elevation (17, 25, 28–31).

This creates a clinical puzzle: neuroimaging can be normal or only subtly abnormal despite markedly elevated pressure (32). This contrasts with other CNS infections that more often display abnormalities that track with a dominant intracranial hypertension mechanism (hydrocephalus, diffuse edema, or mass effect) (33–35).

Because intracranial compliance can evolve over illness, ICP (and CSF opening pressure) should be treated as a dynamic variable rather than as a single baseline value. Accordingly, opening pressure trajectories vary substantially in the first weeks of treatment. In a prospective cohort of 1,443 patients treated for HIV-associated CME, CSF opening-pressure patterns clustered into five phenotypes: persistently elevated (19%), normalizing (35%), fluctuating (8%), delayed elevation (10%), and persistently normal (28%) (11). These heterogeneous pressure trajectories suggest that more than one process can raise ICP in CME, and that both organism properties (e.g., fungal and capsule burden) and host status (e.g., timing of treatment, immune context) may be responsible for these phenotypes.

CME management guidelines emphasize the importance of therapeutic CSF drainage via LPs, which often produce rapid symptomatic improvement (1, 36). This is consistent with an outflow-limited process in which CSF volume is reduced transiently by drainage, but pressure re-accumulates when outflow resistance remains high. The need for repeated drainage over days to weeks is more compatible with a continuously regenerated pressure load (persistent or worsening outflow resistance) than with a purely transient process.

One way to assess outflow resistance is via a constant infusion manometric test, which involves infusing saline at a steady rate into the spinal canal and measuring the resulting pressure change. In a 1968 study, this test was performed on a patient with CME, diagnosed with serologic testing available at the time (37). Compared to 11 control subjects, there was an abnormal pressure rise during infusion, consistent with increased resistance to CSF outflow/absorption. Although supportive of an outflow-resistance mechanism, this evidence is limited by being a single case and by the incomplete ability to localize the site of impaired efflux. Together, these bedside patterns argue that understanding CME-associated intracranial hypertension requires close attention to CSF flow, exchange, and efflux. Accordingly, we next review CSF pathway anatomy and how cryptococcal pathology localizes to these structures.

## CSF PATHWAYS AND CRYPTOCOCCAL LESION TOPOGRAPHY

**Fig 1:**
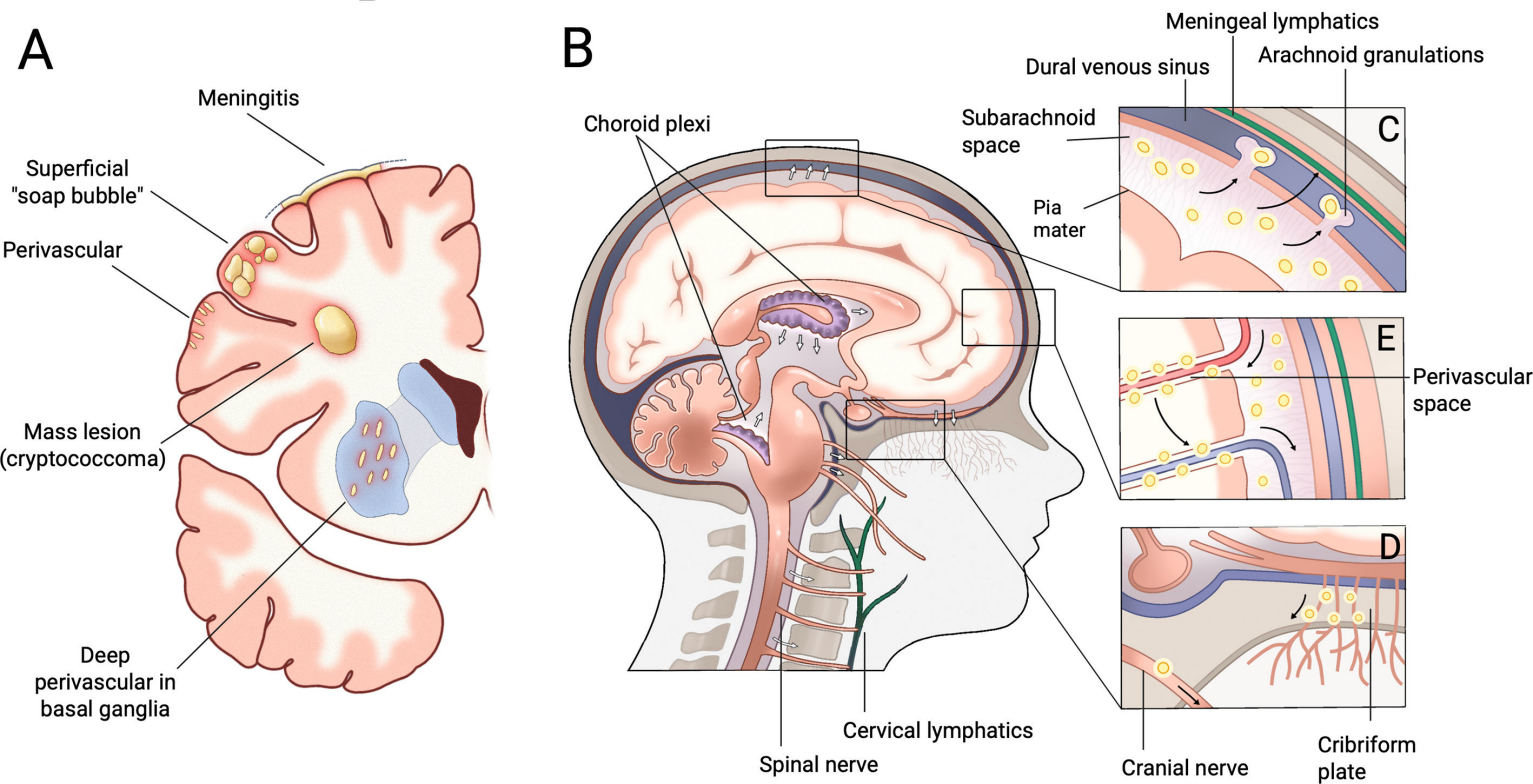
(**A**) Coronal section displaying common gross lesion patterns in cryptococcal meningoencephalitis. Adapted with permission from reference 38. (**B**) Overview of CSF dynamics and clearance. Major CSF outflow routes include (**C**) arachnoid granulations and meningeal lymphatics and (**D**) drainage along cranial nerve sheaths, including olfactory nerve pathways across the cribriform plate to the cervical lymphatics. (**E**) Proposed glymphatic clearance pathways in which CSF enters the brain from arterial perivascular spaces via astrocytic end feet, mixes with interstitial fluid, and exits along perivenous spaces. Yellow circles indicate sites where cryptococcal yeast and cryptococcal capsular polysaccharide have been observed on pathology. Adapted with permission from reference 39.

CME is commonly labeled as a meningitis, but pathology frequently involves both the meninges/subarachnoid space and the underlying brain parenchyma, forming a set of recurring lesion “archetypes” summarized by Sorrell and Davis (38) (Fig. 1A). Below, we summarize the normal CSF compartments and drainage routes most relevant to pressure homeostasis and then describe how cryptococcal organisms and capsular polysaccharide localize to, and may disrupt, these same structures.

### CSF turnover and the subarachnoid compartment

In adults, the total CSF volume is renewed several times daily, such that sustained ICP elevation can arise when CSF absorption/efflux is impaired even without major changes in production (40). After production within and circulation through the ventricular system, CSF enters the subarachnoid space, where CSF opening pressure is measured clinically (39). The subarachnoid space lies between the arachnoid mater and the pia mater. Beneath the pia, the glia limitans forms the outer boundary of neural tissue and is composed of astrocytic end feet (41).

The most common CME lesion pattern is subarachnoid meningitis with cryptococcal cells (and variably inflammatory cells depending on host immune status) within the subarachnoid space, and occasionally, the subpial space (Fig. 1A) (30, 31). Subarachnoid involvement may be diffuse over the cerebral surface or more localized, often involving the base of the brain (38).

A distinctive feature of CME is the high organism burden in CSF coupled with relatively large cell size, with additional burden contributed by abundant shed capsular polysaccharide (3, 42). Quantitative CSF cultures in HIV-associated CME have a reported median of ~10^5^ CFU/mL but can range from approximately 10^2^–10^7^ CFU/mL (43). For comparison, reported tuberculous meningitis bacillary counts in CSF are lower, approximately 10^2^–10^3^ CFU/mL, while bacterial meningitis spans a wide range (~10^3^–10^9^ CFU/mL), though the cells are smaller (44, 45). Typical bacterial cocci have a diameter of approximately 0.5–2 µm (volume of 0.065–4.19 µm^3^, assuming sphericity), whereas encapsulated cryptococcal cells measured in CSF have been reported with a median total diameter of 16.1 µm (volume of ~2,100 µm^3^) (42, 46, 47). It is possible that the relatively large infectious biomass in the CSF and brain parenchyma during cryptococcal infection directly contributes to reduced intracranial compliance due to the volume they occupy.

The combination of large fungal biomass and polysaccharide motivated the hypothesis that increased bulk CSF viscosity might contribute to intracranial hypertension (16). Yet, direct testing of patient CSF found only minimal viscosity increases relative to water, arguing against bulk CSF viscosity as a dominant mechanism (42). A remaining, testable possibility is that at CSF efflux sites, cryptococcal polysaccharide (and/or organisms) could accumulate and increase effective outflow resistance even when lumbar CSF viscosity appears near normal.

### Perivascular spaces: CSF-interstitial exchange and CME lesions

As vessels traverse from the subarachnoid space into brain tissue, they are accompanied by perivascular spaces—anatomical channels between the vessel wall and the glia limitans that persist to the capillary level and then reappear along post-capillary venules as they exit the parenchyma (Fig. 1E) (41). These perivascular compartments are central to the “glymphatic” models of CSF-interstitial fluid exchange, in which CSF enters periarterial spaces, exchanges with interstitial fluid, and then returns along perivenous pathways, in a process influenced by aquaporin-4 localization on astrocytic end feet (48). While the quantitative details of this system remain actively debated, there has been increased study in how disruption of this system could result in neurovascular disease (49).

Cryptococcal accumulation in these perivascular spaces is a common lesion type on pathology (Fig. 1A) (31, 38). Small superficial perivascular lesions contain yeast and inflammatory cells between the vessel endothelium and glia limitans, consistent with cryptococcal entry and accumulation within perivascular spaces after vascular traversal (38). Larger, deep perivascular lesions can have a pseudocystic (“soap bubble”) appearance due to filling with cryptococcal organisms and shed polysaccharide, and on MRI, these may present as enlarged Virchow-Robin spaces that have been associated with worse neurologic outcome (Fig. 1A) (31, 50, 51).

Because perivascular spaces are implicated in CSF-interstitial fluid exchange, widespread filling and enlargement of these spaces in CME raise the testable possibility that cryptococcal organisms cause “perivascular obstruction” and impaired recirculation/clearance that could reduce effective intracranial compliance and worsen ICP dynamics (49). Experimental work in pneumococcal meningitis demonstrates that neuroinflammation can disrupt glymphatic function through detachment of astrocytic end feet from the vascular wall and mislocalization of aquaporin-4, with associated impairment of solute clearance (52). Although potential disruption of the glymphatic system and its role in CME remain untested, the unique deposition and proliferation of cryptococcal cells in these spaces that have more recently become appreciated for their role in CNS homeostasis requires further study.

### CSF efflux routes and pathology

CSF ultimately leaves the subarachnoid space through efflux pathways, and modern evidence supports a distributed drainage system rather than a single dominant route (53). The classic model emphasizes efflux into the venous system via arachnoid villi and granulations projecting into dural venous sinuses, though their quantitative contribution and direct *in vivo* evidence for bulk transport remain debated, and they are absent in certain life stages and in certain species, including mice (Fig. 1C) (53–55). Complementary work supports substantial CSF efflux through lymphatic-associated routes, including traversal along the cribriform plate to nasal lymphatics, perineural routes along exiting cranial/spinal nerves, and meningeal lymphatics in the dura (Fig. 1D) (53, 54, 56). These drainage sites are therefore plausible sites where cryptococcal organisms and shed polysaccharide could increase effective CSF outflow resistance.

Interestingly, there is substantial overlap in these sites and accumulation of cryptococcal organisms on pathology. Human postmortem data (*n* = 5) showed cryptococcal yeasts and free polysaccharide within arachnoid granulations with architectural disruption and inflammatory infiltrates, with correlations between opening pressure and yeast burden within the granulations (29). Additional candidate efflux site involvement includes cryptococcal cells within the optic nerve sheath and organism burden along olfactory/perineural pathways consistent with spread in the direction of CSF egress through the cribriform region (57, 58). To date, no studies have assessed whether cryptococcal cells or products localize to meningeal lymphatics, but their role in CSF absorption has only recently been appreciated (59). Experimental support for the physiological importance of these routes comes from animal work in which blocking cribriform-plate CSF transport increased CSF outflow resistance and raised ICP (60). Overall, these observations are supportive of an outflow-resistance model but should be interpreted cautiously considering small sample sizes and the correlative nature of the pathological findings.

## MECHANISM SYNTHESIS: WHY ICP RISES IN CME

**Fig 2:**
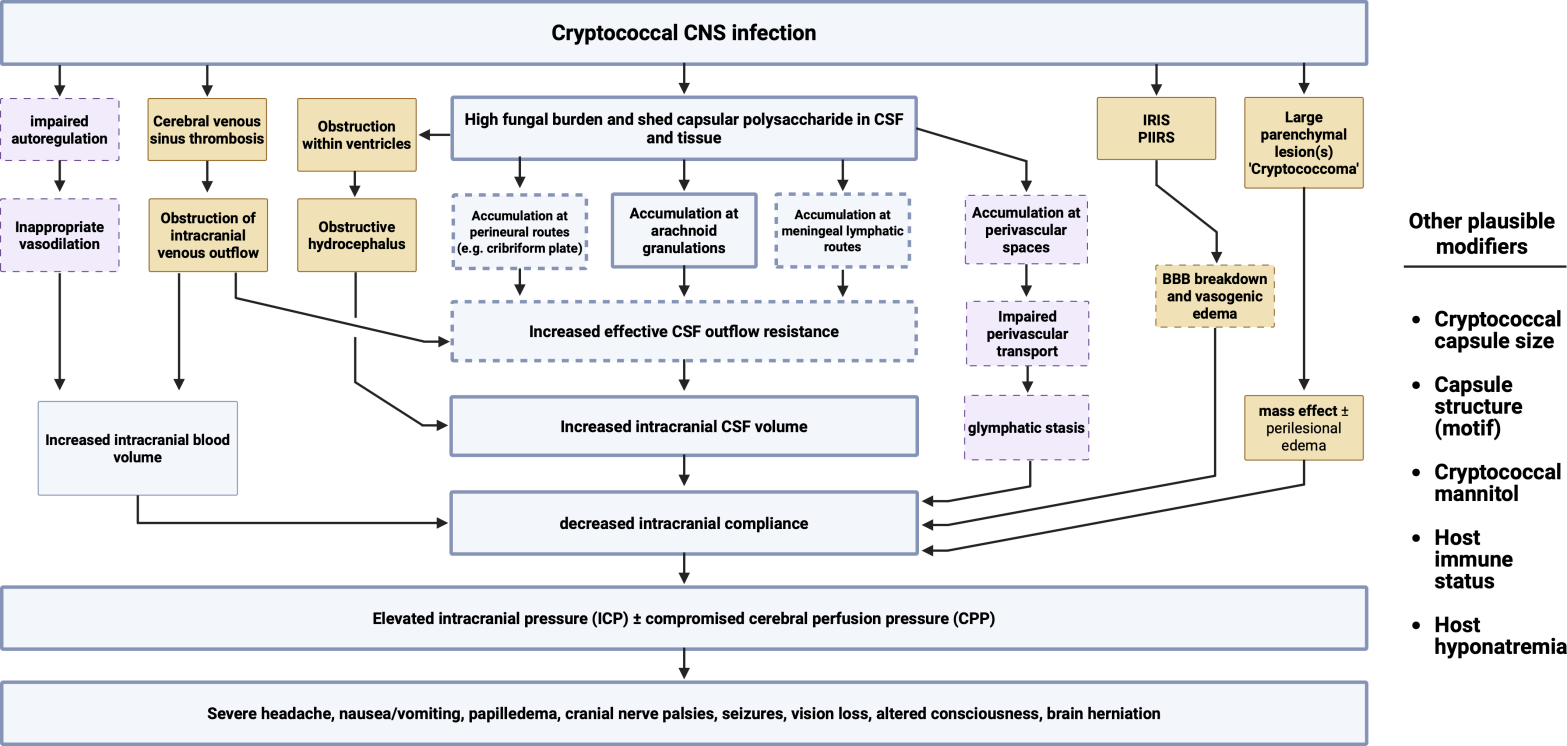
Proposed pathophysiology of intracranial hypertension development in cryptococcal meningoencephalitis. Steps with minimal evidence and requiring further investigation have a dashed outline. Modifying mechanisms are in purple boxes. Mechanisms that likely drive intracranial hypertension in a significant minority of patients are in orange.

We proceed from the working hypothesis that intracranial hypertension in CME most often reflects increased effective CSF outflow resistance, distributed across multiple efflux routes rather than a single fixed obstruction (Fig. 2). This framework extends the classic “arachnoid granulation obstruction” model by incorporating contemporary evidence that CSF efflux can occur via multiple routes, including arachnoid granulations, perineural routes (e.g., cribriform/olfactory), and meningeal lymphatic drainage. Potential modifiers include cryptococcal phenotypes (e.g., capsule architecture and aggregation), host immune status, host osmotic status, and disruption of perivascular transport (“glymphatic”) function. Less commonly, intracranial hypertension is driven primarily by distinct processes that likely require different diagnostic and management approaches.

### High pressure with minimal ventriculomegaly

A distributed outflow-resistance model can explain the pressure-imaging discordance. If resistance increases across multiple efflux routes, subarachnoid pressure can rise without a sustained ventricular-subarachnoid pressure gradient and without ventriculomegaly. As previously suggested by Denning et al., ventricular enlargement is not a necessary consequence of elevated pressure if the principal sites of resistance are downstream of both ventricles and the subarachnoid compartment (16). If absent ventriculomegaly reflects a limited ventricular-subarachnoid pressure gradient, then markers of efflux-site burden should correspond more strongly with opening pressure than ventricular size.

A second, non-exclusive explanation is reduced intracranial compliance, in which small increases in intracranial volume produce large rises in pressure, making ventricular size an unreliable proxy for the severity of intracranial hypertension. In CME, organism and polysaccharide accumulation within subarachnoid and perivascular compartments could plausibly reduce effective compliance, such that substantial pressure elevation occurs without noticeably enlarging the ventricles on imaging. These mechanistic considerations help explain why current care relies heavily on direct CSF pressure/volume management rather than imaging surrogates or broadly effective adjunctive medications.

### Clinical implications for ICP management

Intracranial hypertension in CME is managed primarily with serial LPs, which are associated with improved short-term survival even when baseline opening pressure is not markedly elevated (2, 10, 61, 62). Current guidelines emphasize measuring opening pressure and repeated CSF drainage to reduce pressure toward <20 cmH_2_O (or by ~50% if extremely high) and considering temporary lumbar drainage or ventriculostomy when daily LPs are required, or pressures remain refractory (1, 36). However, repeated invasive procedures can be difficult to implement in high-burden, resource-limited settings, reinforcing the need for less invasive adjunctive strategies grounded in pathophysiology (63–65).

Adjunctive pharmacologic approaches borrowed from other intracranial hypertension syndromes have not translated well: a placebo-controlled acetazolamide trial was stopped early because of excess adverse events and metabolic derangements, and adjunctive dexamethasone led to more adverse events and slower fungal clearance with worse outcomes despite faster opening pressure decreases (15, 66, 67). These negative trials strengthen the rationale for mechanism-driven targets (e.g., CSF outflow resistance at efflux sites) rather than empiric “ICP-lowering” therapies developed for different disease processes.

## MICROBIAL VARIABLES

### Organism burden, particle size, and capsule thickness

Across clinical cohorts, higher opening pressure correlated with higher fungal burden and cryptococcal antigen burden, supporting organism/polysaccharide load as an important contributor to intracranial hypertension (8, 13). In HIV-associated CME, *ex vivo* capsule size measured in baseline CSF was associated with higher CSF opening pressure and remained independently associated in multivariable analysis (42). In that cohort, larger capsule size aligned with a relative paucity of CSF inflammation (including lower levels of multiple cytokines), compatible with a model where capsule enlargement both impairs clearance and contributes to pressure development. Mechanistically, size-dependent lodging of cryptococcal cells has been demonstrated in small brain vessels, raising the related possibility that size and capsule thickness could also influence retention at CSF efflux sites (68–70).

### Phenotypic switching, aggregation, and capsule architecture

Experimental data further support the idea that microbe phenotype influences ICP. In a rat model, phenotypic switch variants differed in their capacity to elicit increased ICP, with the mucoid (larger-capsule) variant producing elevated ICP without ventriculomegaly, whereas a smooth variant did not (71). CSF from mucoid-variant-infected rats contained more aggregated clumps of organisms/polysaccharide and showed greater glucuronoxylomannan (GXM) immunoreactivity, including in perivascular regions (71). Because the mucoid variant also produced higher CSF fungal burden and greater polysaccharide accumulation, these data support a phenotype-linked ICP effect, while also suggesting that phenotypes associated with burden, aggregation, and accumulation at efflux sites may be coupled rather than independent.

Beyond capsule size, capsule chemistry and architecture may influence how readily shed and organism-associated polysaccharide aggregates, how efficiently it is cleared, and whether it preferentially accumulates at specific CSF drainage sites. The major capsular polysaccharide, GXM, can vary in conformational “motifs,” and strains may differ in motif composition with potential virulence implications (4, 72). A testable hypothesis is that specific capsule architectures promote aggregation and/or preferential trapping at efflux sites, thereby increasing effective outflow resistance and worsening intracranial hypertension even at similar quantitative fungal burdens.

### Cryptococcal products and extracellular fluid accumulation

Although diffuse edema is not typically observed in many CME cohorts, cryptococcal products have been investigated as potential contributors to extracellular fluid accumulation in the brain. Early studies by Hirano et al. reported that intracerebral injection of purified cryptococcal polysaccharide in a rat model could cause the accumulation of extracellular fluid in brain parenchyma (73). Future studies will need to re-examine these observations using preparations that better reflect the quantities and structural features of polysaccharides present in patients and that distinguish local tissue effects from mechanisms that would be expected to raise global ICP.

Cryptococcal yeasts are also known to produce mannitol, an osmotic agent that—if generated in sufficient concentration within CSF or brain compartments—could theoretically contribute to increased water content and ICP elevation. In a rabbit model, mannitol was produced *in vivo* and detected in CSF (74). Follow-up work detected mannitol in 19/21 patient CSF samples, but found no correlation with cryptococcal antigen titer or CSF opening pressure (75). Additional studies in experimental models are needed to determine whether cryptococcal mannitol reaches concentrations sufficient to exert meaningful osmotic effects *in vivo* and can cause increased brain water content.

Currently, evidence that GXM or other cryptococcal products can impact cellular-level fluid handling is lacking and requires further investigation. Cryptococcal extracellular vesicles are secreted by cryptococcal cells and carry virulence-associated cargo, including GXM, proteins, lipids, and RNA molecules (76). Known to modulate host cell functioning, they could provide a transport mechanism by which concentrated cryptococcal products could remodel host cellular function and influence intracranial dynamics, but this remains an unproven testable hypothesis.

### Direct vascular effects of cryptococcal products

In addition to outflow-resistance mechanisms, cryptococcal products may plausibly influence ICP through vascular physiology. There is evidence that GXM can sensitize arteries to acetylcholine and promote vasodilation, which could plausibly increase intracranial blood volume (77). Impairment of cerebrovascular autoregulation is well described in other CNS infections, such as experimental pneumococcal meningitis (78, 79). Clarifying whether (and when) cerebrovascular dysregulation contributes meaningfully to CME will likely require studies that pair fungal/polysaccharide measurements with direct assessments of cerebral vascular autoregulation and venous physiology, complemented by experiments testing the impact of GXM (and other cryptococcal products) on vascular tone. Whether vasoactive medications meaningfully modify ICP in CME remains unknown, and current clinical evidence does not support their routine use as mechanism-based adjuncts for pressure control.

## HOST VARIABLES

### Immune state and inflammatory context

Host immune state likely influences intracranial hypertension phenotypes, but across studies comparing HIV-associated versus HIV-negative CME, findings are inconsistent regarding whether intracranial hypertension is more common in one group (80–82). This heterogeneity may reflect differences in the timing of opening pressure measurement, antifungal regimens, and/or the timing of antiretroviral therapy. In at least one cohort, lower peripheral CD4 counts, lower CSF lymphocyte counts, and higher levels of CSF TH1 cytokine levels were associated with intracranial hypertension (83). It is also possible that increased cellularity in the CSF due to inflammation could contribute to CSF resorption impairment, as inflammatory infiltrate has been found within patient arachnoid granulations together with yeast and free polysaccharide (29). Yet the pattern of markedly elevated opening pressure despite limited CSF pleocytosis, most prominent in patients with HIV-associated CME, suggests that inflammatory cell burden alone is unlikely to account for the CME ICP phenotype (42).

### Osmotic disturbances (hyponatremia)

A different host factor that might contribute to intracranial hypertension is hyponatremia. When hyponatremia is accompanied by low serum osmolality, it can promote cerebral water uptake, potentially amplifying intracranial hypertension even if it is not the primary driver (84). A prospective cohort of adults with HIV-associated CME suggests that hyponatremia is common in CME and associates with higher opening pressures and worse outcomes (85). Because the study did not include the testing needed to distinguish between the syndrome of inappropriate antidiuretic hormone secretion and cerebral salt wasting, the precise mechanism of hyponatremia could not be determined, although the authors hypothesized that severe CNS cryptococcosis was an important contributor (85). Severe CNS disease and ICP elevation can trigger neuroendocrine salt/water disturbances, while hyponatremia may, in turn, worsen cerebral swelling. Because the relationship can be bidirectional, hyponatremia is best treated as a clinically important modifier rather than a unifying explanation, especially given the frequent absence of diffuse edema on imaging in many CME cohorts.

## ALTERNATIVE DOMINANT MECHANISMS

Although increased CSF outflow resistance could explain many cases of intracranial hypertension in CME, alternative dominant mechanisms occur in a minority of patients and are critical to recognize because they are likely to change both procedural risks, such as risk of herniation, and management priorities.

### Mass effect (cryptococcoma)

Space-occupying parenchymal lesions can elevate ICP by reducing intracranial compliance and creating pressure gradients that increase herniation risk (86). In CME, mass-like lesions are often labeled “cryptococcomas,” a term used variably in the literature. On histology, these lesions may appear as gelatinous yeast-rich areas with minimal inflammation or as true granulomatous masses with more prominent host inflammatory response (87). Intracranial mass lesions are present in only a minority of CME patients at baseline imaging, and cohort data showing substantially higher cryptococcoma frequency in *C. gattii* than *C. neoformans* without a corresponding difference in mean/median opening pressure suggest cryptococcomas are unlikely to be a dominant driver of intracranial hypertension in most patients (88). Cryptococcomas are best treated as an important exception that is clinically relevant for focal complications and procedural risk, rather than a primary explanation for the common CME phenotype of marked opening-pressure elevation without overt mass effect.

### Hydrocephalus

While ventriculomegaly is not typical in many CME patients with high opening pressure, some patients develop hydrocephalus, including obstructive forms (89, 90). When hydrocephalus is clinically significant, CSF diversion (e.g., ventriculoperitoneal shunting) may be required and has been reported as feasible and effective (91).

### Venous outflow impairment (cerebral venous sinus thrombosis)

Reduced cerebral venous drainage can raise ICP by increasing intracranial blood volume and by secondarily impairing CSF absorption through elevated venous sinus pressure. In cerebral venous sinus thrombosis (CVST), clotting can occur in these large veins, blocking blood drainage. A recent study reported a CVST incidence of 5.6% in immunocompetent CME, comparable to the incidence seen in bacterial and viral meningitis (92). This suggests that while CVST may not be the primary mechanism of intracranial hypertension seen in most patients, it can be a rare but significant cause during CME.

### Immune reconstitution inflammatory syndrome and post-infectious inflammatory response syndrome

Post-infectious inflammatory response syndrome (PIIRS) and immune reconstitution inflammatory syndrome (IRIS) represent key contexts in which inflammation can become a dominant contributor to intracranial hypertension after antifungal therapy and/or immune recovery (93). In these syndromes, intracranial hypertension may reflect a mechanistically distinct inflammatory state (e.g., vasogenic edema following inflammatory blood-brain barrier leakage), rather than a primarily organism/polysaccharide-mediated outflow resistance (94). This framing is consistent with the clinical observation that corticosteroids may be beneficial in PIIRS/IRIS-associated neurologic deterioration, despite being harmful as routine adjunctive therapy in HIV-associated CME more broadly (95–97).

These exceptions underscore an important point: while inflammation, venous outflow impairment, hydrocephalus, or mass effect can dominate in select contexts, the most common CME presentation remains sustained, high opening pressure that behaves like a CSF outflow-limited state.

### Conclusion: why cryptococcus?

The ability for *C. neoformans* to elicit large, sustained elevations in ICP raises a basic question: what organism features (interacting with the host) make intracranial hypertension so common in CME? *C. neoformans* is distinctive among pathogenic eukaryotic microbes in combining a large polysaccharide capsule with copious shedding of capsular polysaccharide into tissues, including CSF (3, 4). This creates a CSF environment that contains not only many viable yeasts but also abundant extracellular polysaccharides that can persist and distribute within CSF pathways. In parallel, cryptococcal cells are large at baseline and can become substantially larger with encapsulation. Pathology has shown that these organisms, whether through tropism or passive transport with CSF flow, can accumulate at CSF efflux sites where they are able to survive, proliferate, and disrupt normal architecture. In the last decade, there have been major advancements in our understanding of both CSF dynamics and cryptococcal biology within the host, making it possible for future work to better define what host and microbe factors lead to this complication. It is our hope that research that pinpoints where outflow resistance emerges and describes how cryptococcal burden, capsular polysaccharide, and host responses remodel CSF efflux sites will lead to effective adjunctive therapeutics that will lower the morbidity and mortality of this infection.

Future research priorities are as follows:

- Map and quantify cryptococcal cells/polysaccharide at defined CSF efflux sites (e.g., perineural/cribriform routes, meningeal lymphatics) and test whether local burden predicts measured opening-pressure trajectories.
- Build experimental systems that directly measure CSF outflow resistance and efflux to establish causality.
- Define which cryptococcal phenotypes (capsule size/architecture, aggregation) and host factors (immune state/IRIS context, osmotic state) shift the system into high-resistance, low-compliance conditions.
- Test whether perivascular space (“glymphatic”) disruption in CME measurably alters compliance/pressure dynamics.

## AFTER EPUB

*[This article was published on 13 April 2026 with errors in the Fig. 1 legend. The legend was corrected in the current version, posted on 13 April 2026.]*
